# Factors Associated with Dental Fear and Anxiety in Children Aged 7 to 9 Years

**DOI:** 10.3390/dj7030068

**Published:** 2019-07-01

**Authors:** Andreas Dahlander, Fernanda Soares, Margaret Grindefjord, Göran Dahllöf

**Affiliations:** 1 Department of Pediatric Dentistry, Public Dental Service, 151 73 Södertälje, Sweden; 2 Center for Pediatric Oral Health Research, Pediatric Dentistry, Public Dental Service, Eastman Institute, SE-113 24 Stockholm, Sweden; 3 Department of Dental Medicine, Division of Orthodontics and Pediatric Dentistry, Karolinska Institutet, 171 77 Stockholm, Sweden; 4 Center for Oral Health Services and Research (TkMidt), Mid-Norway, 7030 Trondheim, Norway

**Keywords:** dental anxiety, children, dental fear, longitudinal study

## Abstract

The aim was to investigate changes in dental fear and anxiety (DFA) and verify factors associated with DFA in children. A longitudinal cohort study that included 160 children aged 7 years was carried out. A questionnaire was completed by parents at two time points and evaluated the immigrant background, maternal education, whether the child had ever had toothache, and whether the parents had dental fear. The oral clinical examination evaluated decayed, extracted, and filled primary teeth (deft). The children’s fear survey schedule dental subscale (CFSS-DS) was used to assess the dental fear of the children. Multilevel mixed-effects logistic regressions analyses were used. The CFSS-DS found that 7% of the children had dental fear at age 7 and mean CFSS-DS was 22.9. At 9 years of age, 8% reported dental fear and the mean increased to 25.4. Parental dental fear, experience of toothache, and report of painful dental treatment and caries development between 7 and 9 years of age were factors that were significantly related to development of DFA. There was a change in DFA between 7 and 9 years of age. Dental fear and anxiety is a dynamic process in growing individuals and is significantly related to painful symptoms and experiences of dental care as well as parental dental fear.

## 1. Introduction

Dental fear and anxiety (DFA) is one of the major challenges in pediatric dentistry [1]. The prevalence is estimated to approximately 9% [2]. Using the children’s fear survey schedule-dental subscale (CFSS-DS), 6.7% of a Swedish sample were assessed as being fearful [3]. DFA is a common reason for avoiding dental treatment, which over time, may result in deteriorated oral health [4]. DFA among children has a complicated and multifactorial etiology [5]. Several interacting factors, personal as well as environmental, contribute to the development of fear and anxiety in a dental care situation [6]. Psychological factors such as shyness and general fearfulness or immaturity have previously been investigated and found to be important [7]. Cognitive ability [7] as well as transmission of negative attitudes from parents or others are also pathways of DFA acquisition [8]. Several studies have shown an association between parental and child DFA [8,9]. Culture is most likely to influence DFA [10]. One study in a multicultural area on the outskirts of Stockholm showed that DFA in seven-year-old children is more common among those with a foreign background compared to those of Swedish origin [11]. Experience of dental treatment also increases the risk for DFA [12,13]. A study by Raadal et al. found that 68% of the children with high dental anxiety had more than five carious lesions at five years of age [14].

The age of the child is important when coping with stressful situations such as dental treatments [12]. Cross-sectional studies assume that DFA decreases with age [15]. However, few studies have actually investigated changes in DFA over time [13,16]. Longitudinal studies have found that DFA increases with age in young schoolchildren [8,13].

This study investigated changes in DFA over a two-year period using CFSS-DS in seven- to nine-year-old children. The hypothesis was that dental treatment contributes to development of dental fear.

## 2. Materials and Methods

### 2.1. Subjects and Procedures

A longitudinal study on children aged 7–9 years was conducted from 2011 to 2013. The target population of the study at baseline was 7-year-old children (n = 196) who were scheduled for their annual dental check-up at the Public Dental Service clinic in Södertälje. In Sweden, children are entitled to free bi-annual dental examinations and treatment from 3 to 19 years of age. Children with cognitive disabilities were excluded this study, because of the difficulties measuring DFA in this group. This study included all children aged 7 years seen at the Public Dental Service clinic in Södertälje in 2011 and who returned to the consultation in 2013 (9 years). The sample calculation was carried out a posteriori, and for the analysis conducted in the present study, it is possible to detect as significant odds ratio higher than 1.5, with 95% confidence interval, and statistical power higher than 80%.

### 2.2. Ethics Approval and Consent to Participate

The Human Research Ethics Committee at Karolinska Institutet approved the protocol (Daybook no. [Dnr.] 2011/443-32, 8/4 2011 and 2017/162-32), 10 February 2017 and all parents or guardians of the children signed informed consent forms.

### 2.3. Measures

At the check-up, parents were asked to fill in a form with questions regarding ethnicity. To be classified as having an immigrant background, either the child was born outside Sweden or the child was born in Sweden with two foreign born parents. Furthermore, maternal education level (≤8 years of education, >8 years of education), whether the child had ever had toothache (no, yes), parental attendance pattern (non-regular ≥ 1 missed appointment, regular), and parental dental fear (no, yes) were measured. The examiner assessed patient cooperation according to the Frankl behavioral rating scale.

The dentist evaluated gingivitis (no, yes), plaque (no, yes), and dental caries in the primary and permanent teeth, which was classified as number of decayed (d), extracted (m), and filled (f) primary teeth, and decayed (D), missing (M), and filled (F) permanent teeth. All children in the study were examined at the same clinic Two dentists were assigned to examine the children and collect the data.

The parental version of the CFSS-DS was used to assess the dental fear of the children. The CFSS-DS form was filled in by parents on behalf of their children. This 15-item instrument uses a 5-point Likert scale for item scores ranging from 1 (not afraid at all) to 5 (very afraid), giving a total range of 15–75. Scores equal to or above 38 have been suggested to indicate high fear of going to the dentist among children; thus, we chose 38 as the cut-off score for DFA [17,18].

The children were re-examined at 9 years of age and parent’s children filled in the same questionnaire as they had 2 years previously. Number of injections and extractions made during this 2-year period was determined from the dental records, as was number of new carious teeth in the permanent and primary teeth.

### 2.4. Statistical Analyses

All statistical analyses were conducted in STATA 13 for Windows (StataCorp., College Station, TX, USA, 2013. Stata Statistical Software: Release 13. College Station, TX, USA: StataCorp LP). For descriptive purposes, crude and relative frequencies are presented. Chi-square or Fisher’s exact test was used to examine the significance of observed differences. The Wilcoxon test evaluated whether the mean of the each CFSS question differed between monitoring periods while kappa evaluated the stability (tracking) of high dental fear in the children over time.

Multilevel mixed-effects logistic regressions were used to analyze which factors were longitudinally associated with high dental fear. First, several analyses were performed (crude model) to determine which factors were associated with high dental fear. All final models were adjusted for the sex and nationality of the child. Results are presented as odds ratios and 95% confidence intervals.

## 3. Results

At age nine years, 160 children were re-examined; 18% did not attend. The most common reasons were having moved from the area or recall outside of the study period. The dental health of the drop-outs is unknown, but of the examined variables, only parental dental fear was significantly higher in the drop-out group compared to those examined at nine years of age.

Table 1 presents the characteristics of the child participants. At the seven-year-old examination, 39% of the children had an immigrant background. From the CFSS-DS, 7% of children at age seven suffered from DFA, with a mean CFSS-DS of 22.9 ± 6.9. At nine years of age, 8% reported DFA and the mean increased to 25.4 ± 8.7 (*p* < 0.001). Thirty per cent of the children were diagnosed with dental caries in the primary dentition at seven, and 43% at nine years of age.

We documented all dental care between seven and nine years of age. At nine years, 31% of the children had developed carious teeth in the primary dentition, and 12% in the permanent dentition; 14% had had extractions; and 19% had received injections with local anesthesia.

Table 2 shows potential risk factors for development of DFA defined as CFSS-DS ≥ 38 in a univariate analysis at seven and nine years of age. Variables related to the experience of pain and discomfort as well as variables related to dental treatment were associated with DFA. At nine years of age, such variables included development of dental caries between seven and nine (*p* = 0.012), experience of dental caries in primary dentition (*p* = 0.045), injections with local anesthesia (*p* = 0.042), and extractions (*p* = 0.001).

**Figure 1:**
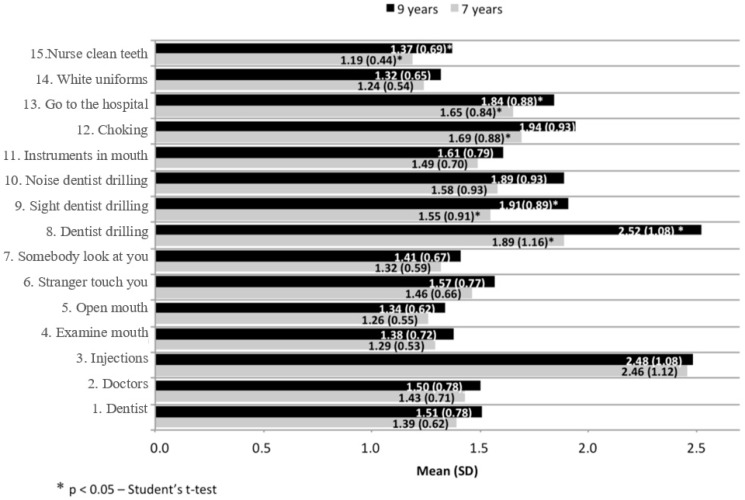
Mean of the responses for the items on the children’s fear survey schedule-dental subscale (CFSS-DS) at ages seven and nine years. Y-axis: Do you fear.

Figure 1 presents the results of the 15 items on the CFSS-DS; fear of injection scores were high at seven years of age (2.5 ± 1.1), and both fear of injections (2.5 ± 1.1) and fear of the dentist drilling (2.5 ± 1.1) at nine years of age. Five variables increased significantly between seven and nine years of age: Fear of the dentist drilling, of seeing the dentist drill, of choking, of having to go to the hospital, and of the dentist cleaning the teeth.

**Figure 2:**
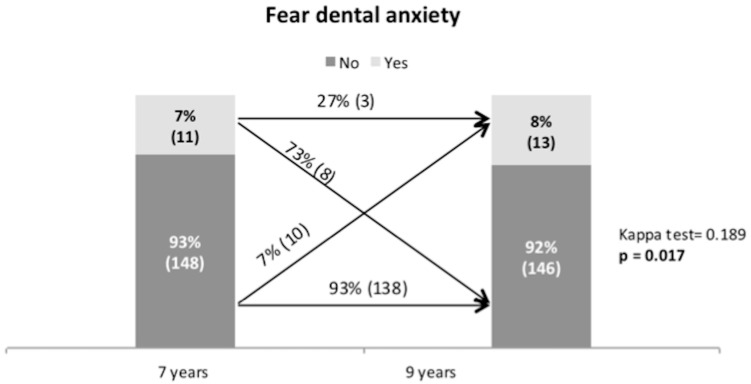
Change in dental fear and anxiety (DFA) between seven and nine years.

Figure 2 shows the change in DFA between seven and nine years of age. At seven years, 7% of the examined children reported a CFSS-DS ≥ 38; the percentage of children reporting DFA was higher at nine years of age when 8% reported high DFA. The majority of children, 73%, who were fearful at age seven, did not report dental fear at nine years of age. On the other hand, 7% of children that did not report DFA at age seven, did so at nine. Of children who were fearful at the age of seven years and did not report dental fear at nine years of age, 12.5% had dental treatment, and of children who become fearful, 30% had dental treatment. As Figure 2 shows, it is not the same individuals who are diagnosed with DFA at seven as at nine years of age, and this difference is significant (*p* = 0.017).

### Multivariate Analyses

We used the CFSS-DS at a cut-off of ≥38 to classify children with DFA. In a first step, we selected the significant univariate-test variables (Table 2) for a univariate multilevel regression in order to identify which variables remained associated with DFA. We ran five models using multivariate multilevel logistic regression. All models were adjusted for gender, maternal education, and immigrant background. In all models, children whose parents reported DFA were 4 times more likely to be diagnosed with DFA Children who reported having had toothache had 4 times higher OR of dental fear (CI 95%: 1.07–13.54). Experience of painful dental treatment increased the OR of the child’s chance to be diagnosed with DFA by 5 times (CI 95%: 1.14–23.10), and children who had developed caries between ages seven and nine had a 4 times higher OR of having fear dental (CI 95%: 1.04–13.18, Table 3).

## 4. Discussion

This study diagnosed 7% of the seven-year-old children and 8% of the nine-year-old children with DFA. Of the children reporting DFA at seven years of age, 73% did not report DFA at nine. On the other hand, 7% of the nine-year-olds had developed dental fear between seven and nine years of age. Parental dental fear, experience of toothache, painful dental treatment, and caries development between seven and nine year of age were significantly related to the development of DFA between seven and nine years of age.

Few longitudinal studies have been done on the development of dental fear [8]. Most information on the prevalence of DFA in children stems from cross-sectional studies. The Klingberg et al. [17] study on 4- to 11-year-olds used the same CFSS-DS cut-off of ≥38, reporting that 7% of Swedish children exhibited DFA. Wogelius et al. found that 7% of Danish six-year-olds, 5.7% of seven-year-olds, and 4.4% of eight-year-olds reported DFA [19]. It is interesting to note that there has been no observable decline in the prevalence of DFA that parallels the decline in prevalence of dental caries in children. Child populations in Scandinavia are not as homogenous as they previously were, and there are groups of children with high increments of caries in the preschool and school ages [20].

The most striking finding in this study is the transition in DFA between seven and nine years of age. Of children who were fearful at age seven, 73% reported no DFA at age nine, and 7% who were not fearful at seven reported DFA at nine. Tickle et al. (2009) reported similar results using a five-point Likert scale to diagnose DFA [13]. They followed children from five to nine years of age, and 12% developed DFA during these years. Recently, Soares et al. [16] found that 15% of children who reported no DFA at age five had developed DFA at nine. The results of our study provide more conclusive evidence for several transitions regarding DFA in early school ages. DFA may wear off with increasing age and cognitive development, but it may also develop during this period, depending on family factors and factors related to poor oral health and dental treatment [5].

CFSS-DS has 15 items; this study found that fear of injections, of the dentist drilling, and of choking were the items with the highest scores. This is similar to the study by Wogelius et al. (2009) [21], which reported fear of injections, fear of choking, and fear of the dentist drilling as the three items with the highest scores. In our study, fear of the dentist drilling, sight of the dentist drilling, fear of choking, having to go to the hospital, and having the dentist clean your teeth all increased significantly between seven and nine years of age. It is surprising that there was no increase in fear of injections. On the other hand, only 19% of the children had injections during the period and this was the item with the highest score at age seven, the start of the study. Fear of injections often develops due to vaccinations at the doctor’s office [14,22], so it may not be realistic to expect this item to change much.

In the multivariate analysis, we examined factors related to development of DFA between seven and nine years of age using various models that also accounted for confounding factors. All models were adjusted for gender, maternal education, and nationality. We found a significant association with parental DFA, experience of toothache, experience of painful dental treatment, and development of dental caries between seven and nine years of age. First, previous studies [22,23,24] had shown an association between parental DFA and child DFA. It was clear that experience of dental treatment, particularly painful and traumatic experiences, are possible sources of DFA [14]. Raadal et al. (2002) [14] reported that DFA at age 10 years was associated with the experience of caries development and dental treatment between 5 and 10 years of age. One study reported an association between the number of filled surfaces and DFA in 3- to 14-year-olds and another [25], a significant association between number of painful dental experiences between 12 and 18 years of age and DFA.

It is important to realize that this is not a population-based sample of children. The cohort included all children scheduled for a bi-annual dental check-up at one large public dental service clinic. There is a risk that patients and parents with high levels of dental anxiety do not show up for examination or do not want to participate in the study. Another limitation is the use of parental-reported CFSS-DS although there is a moderate correlation between child- and parental-reported CFSS-DS [26]. Strengths of this study include its longitudinal design, use of CFSS-DS as a measure of DFA, and the possibility to study individual items in the CFSS-DS. The clinical implications of this study are that patients need psychological preparation for potentially painful or difficult dental procedures. An interesting possibility is the use of guided self-help cognitive behavioral therapy, using a booklet or web-based program,, exposing the child to situations they will experience in the clinic [27]. CBT is a structured and brief psychological treatment based on a combination of psychoeducation, exposure, and homework exercises [28]. The treatment approach is effective in increasing a patient’s ability to manage dental procedures, increase self-efficacy, and reduce fear related to specific dental procedures [29].

## 5. Conclusions

In conclusion, this study showed an increased prevalence of dental fear and anxiety between seven and nine years of age. Furthermore, that development of new carious lesions, experience of toothache, and extractions were the most significant risk factors for development of DFA. Dental treatment should focus beyond the prevention of dental caries, on the psychological aspects that the treatment can cause, thus, preventing painful and traumatic experiences.

## Figures and Tables

**Table 1:** Characteristics of children at ages seven and nine years.

Variables	Category	Age	
		7 years *n* (%)	9 years *n* (%)
Gender	Girl	79 (40)	63 (39)
	Boy	117 (60)	97 (61)
Immigrant background	Swedish	118 (60)	96 (60)
	Immigrant	77 (40)	63 (40)
Mother’s education	≤8 years	27 (14)	23 (15)
	>8 years	164 (86)	132 (85)
Parental dental fear	No	150 (79)	124 (77)
	Yes	40 (21)	37 (23)
Parental dental attendance	Irregular	45 (24)	26 (16)
	Regular	145 (76)	135 (84)
Child report of toothache	No	165 (85)	122 (76)
	Yes	29 (15)	38 (24)
Child report of painful treatment	No	176 (91)	142 (88)
	Yes	17 (9)	19 (12)
Child cooperation	Positive	191 (99)	148 (93)
	Negative	2 (1)	11 (7)
Dental fear (CFSS-DS ≥ 38)	No	183 (94)	147 (92)
	Yes	11 (6)	13 (8)
Gingivitis	No	172 (89)	143 (92)
	Yes	22 (11)	13 (8)
Plaque	No	167 (86)	145 (96)
	Visible	27 (14)	6 (4)
Dental caries in permanent teeth	No	185 (95)	134 (83)
	Yes	10 (5)	27 (17)
Dental caries in primary dentition	No	136 (70)	92 (57)
	Yes	58 (30)	69 (43)

**Table 2:** Potential risk factors for dental fear at ages seven and nine years.

Variables	Categories	7 Years			9 Years		
		No Fear	Fear	*p*	No Fear	Fear	*p*
		%	%		%	%	
Sex ^¶^	Girl	94	6	0.742	91	9	0.602
	Boy	95	5		93	7	
Immigrant background ^¶^	Swedish	96	4	0.272	92	8	0.929
	Immigrant	92	8		92	8	
Mother’s education ^¶^	≤8 years	85	15	**0.030**	83	17	0.061
	>8 years	96	4		94	6	
Parental dental fear ^¶^	No	95	5	0.203	94	6	**0.038**
	Yes	90	10		84	16	
Parental dental attendance ^§^	Irregular	96	4	0.652	100	0	0.092
	Regular	94	6		90	10	
Child report of toothache ^¶^	No	97	3	**<0.001**	93	7	0.535
	Yes	79	21		89	11	
Child report of painful treatment ^¶^	No	96	4	**0.001**	94	6	**0.027**
	Yes	76	24		79	21	
Dental caries in permanent teeth ^§^	No	94	6	0.585	92	8	0.889
	Yes	100	0		93	7	
Dental caries in primary dentition ^¶^	No	96	4	0.233	96	4	**0.045**
	Yes	91	9		87	13	
Extractions ^¶^	No	93	7	0.568	95	5	**0.001**
	Yes	90	10		75	25	
Injections ^¶^	No	94	6	0.468	95	5	**0.042**
	Yes	90	10		84	16	
Increase in caries in primary teeth ^¶^	No	95	5	0.276	95	5	**0.012**
	Yes	90	10		84	16	
Increase in caries in permanent teeth ^¶^	No	93	7	0.762	91	9	0.621
	Yes	95	5		95	5	

^¶^ Chi-square test; ^§^ Fisher’s exact test; Bold = *p* < 0.05

**Table 3:** Multilevel logistic regression for the incidence of dental fear and anxiety.

Variables	Crude Model		Model I		Model II		Model III		Model IV		Model V	
	OR	*p*	OR	*p*	OR	*p*	OR	*p*	OR	*p*	OR	*p*
	(IC 95%)		(IC 95%)		(IC 95%)		(IC 95%)		(IC 95%)		(IC 95%)	
**Parental Dental Fear**												
No	1	**0.037**	1	**0.038**	1	**0.038**	1	**0.021**	1	**0.026**	1	**0.027**
Yes	3.17		3.61		3.86		4.05		4.09		3.8	
	(1.07–9.35)		(1.08–12.11)		(1.17–12.67)		(1.23–13.32)		(1.19–14.09)		(1.16–12.40)	
**Child Report of Toothache**												
No	1	**0.019**	1	**0.040**	-	-	-	-	-	-	-	-
Yes	3.86		3.8		-		-		-		-	
	(1.25–11.99)		(1.07–13.54)									
**Child Report of Painful Treatment**												
No	1	**0.003**	-	-	1	**0.033**	-	-	-	-	-	-
Yes	8.12		-		5.14		-		-		-	
	(2.01–32.77)				(1.14–23.10)							
**Dental Caries in Primary Dentition**												
No	1	**0.040**	-	-	-	-	1	**0.060**	-	-	-	-
Yes	3.43		-		-		3.59		-		-	
	(1.06–11.11)						(0.95–13.61)					
**Extractions**												
No	1	**0.036**	-	-	-	-	-	-	1	0.238	-	-
Yes	5.01		-		-		-		2.64		-	
	(1.11–22.56)		-		-				(0.53–13.28)			
**Increase in caries in primary teeth**												
No	1	**0.029**	-	-	-	-	-	-	-	-	1	**0.044**
Yes	3.96		-		-	-	-	-	-		3.7	
	(1.15–13.64)										(1.04–13.18)	

Model I—Parental fear, children toothache, and adjusted (gender, maternal education, and nationality of the child); Model II—Parental fear, painful treatment, and adjusted (gender, maternal education, and nationality of the child); Model III—Parental fear, dmfs, and adjusted (gender, maternal education, and nationality of the child); Model IV—Parental fear, extractions, and adjusted (gender, maternal education, and nationality of the child); Model V—Parental fear, increase in caries in primary teeth, and adjusted (gender, maternal education, and nationality of the child). Bold = *p* < 0.05
